# MicroRNAs, Genomic Instability and Cancer

**DOI:** 10.3390/ijms150814475

**Published:** 2014-08-20

**Authors:** Kimberly Vincent, Martin Pichler, Gyeong-Won Lee, Hui Ling

**Affiliations:** 1 Department of Experimental Therapeutics, the University of Texas MD Anderson Cancer Center, Houston, TX 77054, USA; E-Mails: KYVincent@mdanderson.org (K.V.); MPichler@mdanderson.org (M.P.); 2 Division of Hematology-Oncology, Department of Internal Medicine, Gyeongsang National University Hospital, Gyeongsang National University School of Medicine, Jinju 660-702, Korea; E-Mail: brightree24@gmail.com

**Keywords:** microRNAs, genomic instability, cancer

## Abstract

MicroRNAs (miRNAs) are small non-coding RNA transcripts approximately 20 nucleotides in length that regulate expression of protein-coding genes via complementary binding mechanisms. The last decade has seen an exponential increase of publications on miRNAs, ranging from every aspect of basic cancer biology to diagnostic and therapeutic explorations. In this review, we summarize findings of miRNA involvement in genomic instability, an interesting but largely neglected topic to date. We discuss the potential mechanisms by which miRNAs induce genomic instability, considered to be one of the most important driving forces of cancer initiation and progression, though its precise mechanisms remain elusive. We classify genomic instability mechanisms into defects in cell cycle regulation, DNA damage response, and mitotic separation, and review the findings demonstrating the participation of specific miRNAs in such mechanisms.

## 1. Introduction

The ENCODE project suggests that the majority of the human genome is transcribed into RNA transcripts. However, genes that code for proteins only comprise 2% of the human genome, and cannot fully explain the complexity of physiological and pathological events [1]. In the last decade, non-coding RNA (ncRNA) transcripts not translated into proteins have been shown to participate in cancer initiation, progression and metastasis. So far the most well characterized ncRNAs are a class of short ncRNAs with 19–24 nucleotides, termed microRNAs (miRNAs). The miRNA processing begins in the nucleus, where miRNA genes are transcribed by RNA polymerase II (pol II) into primary miRNA transcripts (pri-miRNAs), which are subsequently polyadenylated and capped. The RNase III enzyme Drosha, along with the DGCR8 protein, cleaves Pri-miRNAs into stem-loop structured precursor miRNAs (pre-miRNAs). Exportin-5 mediates the transport of these pre-miRNAs into the cytoplasm, where their loop is cleaved by Dicer to produce the ~20 nt miRNA duplex. This mature miRNA duplex is unwound by various helicases into a passenger strand (which is subsequently degraded) and a mature guide strand. The mature miRNA is loaded with Argonaute2 (Ago2) into the RNA-induced silencing complex (RISC), and guides RISC to repress gene expression by translational silencing or inducing the degradation of mRNA, via complementary binding to, mostly, the 3' untranslated region of target genes [2].

MiRNAs were first discovered in *Caenorhabditis elegans* by Victor Ambros and Gary Ruvkun’s groups in 1993 [3]. However, the biological functions of miRNAs in human cancer were not appreciated until 2002, when Drs. George Calin and Carlo Croce cloned miR-15a/16-1 from a recurrently deleted chromosomal region lacking the protein culprit in chronic lymphocytic leukaemia (CLL) patients, the most common leukaemia in adults [4]. These two miRNAs were found to be downregulated in around 70% of all the CLL cases examined, and act as tumor suppressors by regulating anti-apoptotic proteins such as BCL-2 and MCL-1 [5]. Subsequently, many other groups identified deregulated miRNA profiles in different types of human cancers, and demonstrated their roles in promoting or inhibiting cancer pathogenesis. Typical examples of the miRNAs involved in human cancer include miR-15a/16-1 and miR-34, which possess tumor suppressor functions, and miR-17-92 cluster and miR-155, which have oncogenic activities [6]. MiRNAs have also been intensively investigated for their potential applications as diagnostic markers, prognostic markers and therapeutic targets for cancer patients [7,8,9].

In the most updated version of the seminal review paper by Hanahan and Weinberg, genomic instability was included as a new entity enabling characteristics of cancer, along with other cancer hallmarks including sustaining proliferative signaling, evading growth suppressors, resisting cell death, enabling replicative immortality, inducing angiogenesis, activating invasion and metastasis, deregulating cellular energetics, and avoiding immune destruction [10]. Genomic instability is defined as a high frequency of mutations within the genome, including changes in nucleic acid sequences, chromosomal rearrangements, or aneuploidy. Although there is still a debate whether genomic instability is a cause or a consequence of carcinogenesis, it is present in nearly all types and stages of human cancers, and affects prognosis and treatment responses of cancer patients. Genomic instability can be in the form of chromosomal instability, microsatellite instability, or significantly heightened levels of mutations at the nucleotide level [11]. The stability of the human genome is maintained by multiple mechanisms such as the cell cycle checkpoint, DNA damage response, mitotic separation machinery; any defects in maintenance of these mechanisms could lead to increased fragility of the genome.

**Table 1:** MiRNAs involved in regulation of genomic instability.

miRNAs	Cancer Type	Targets	Function	Category	Effect	Reference
miR-16	Breast cancer	WIP1	Enhances response to DNA damage	DNA damage response	Prevents genomic instability	[12]
miR-24	Hematopoietic malignancies	E2F2, CDK1, CDK4	Increases cell population in the G1 phase	Cell cycle	Enhances genomic instability	[13]
miR-24	Hematopoietic malignancies	H2AX	Impairs DNA repair	DNA damage response	Enhances genomic instability	[14]
miR-24	Hepatocellular carcinoma	AURKB	Impairs chromosome separation	Mitotic events	Enhances genomic instability	[15]
miR-29	Breast cancer, colon cancer	PIK3R1 and CDC42	Increases p53 stability	DNA damage response	Prevents genomic instability	[16]
miR-34	Colon cancer, hepatocellular carcinoma	CCND1, CCNE2, CDK4, MET, MYC, and SIRT1	Promotes DNA damage repair, or induces apoptosis	DNA damage response	Prevents genomic instability	[17,18,19,20]
miR-96	Osteosarcoma, breast cancer	RAD51	Impairs HR repair	DNA damage response	Enhances genomic instability	[21]
miR-100	Nasopharyngeal cancer	PLK1	Loss of miR-100 promotes mitotic catastrophe	Mitotic events	Prevents genomic instability	[22]
miR-100	Glioblastoma	ATM	Impairs response to DNA damage	DNA damage response	Enhances genomic instability	[23]
miR-101	Glioblastoma	ATM	Impairs response to DNA damage	DNA damage response	Enhances genomic instability	[24]
miR-101	Glioblastoma	PRKDC	Impairs NHEJ repair	DNA damage response	Enhances genomic instability	[24]
miR-103	Hepatocellular carcinoma, osteosarcoma, ovarian cancer	RAD51	Impairs HR repair	DNA damage response	Enhances genomic instability	[25]
miR-106b-25 cluster	Colon cancer	p21	Promotes the G1-to-S transition	Cell cycle	Enhances genomic instability	[26]
miR-107	Hepatocellular carcinoma, osteosarcoma, ovarian cancer	RAD51	Impairs HR repair	DNA damage response	Enhances genomic instability	[25]
miR-122	Hepatocellular carcinoma	CCND1	Upregualtes p53 expression	DNA damage response	Prevents genomic instability	[27]
miR-125b	Head and neck cancer	MXD1	Delays mitotic progression at metaphase	Mitotic events	Enhances genomic instability	[28]
miR-125b	Neuroblastoma	P53	Reduces p53 expression	DNA damage response	Enhances genomic instability	[29]
miR-138	Osteosarcoma	H2AX	Impairs response to DNA damage	DNA damage response	Enhances genomic instability	[30]
miR-148b	Breast cancer	RAD51	Impairs HR repair	DNA damage response	Enhances genomic instability	[31]
miR-155	Colon cancer, breast cancer	MLH1, MSH2, MSH6, TERF1	Promotes microsatellite instability; impairs telomere integrity	DNA damage response	Enhances genomic instability	[32,33]
miR-182	Colon cancer, breast cancer	BRCA1	Impairs response to DNA damage	DNA damage response	Enhances genomic instability	[34]
miR-193b	Breast cancer	RAD51	Impairs HR repair	DNA damage response	Enhances genomic instability	[31]
miR-193b	Breast cancer	BRCA1 and BRCA2	Impairs HR repair	DNA damage response	Enhances genomic instability	[31]
miR-210	Breast cancer, hepatocellular carcinoma	RAD52	Impairs HR repair	DNA damage response	Enhances genomic instability	[35]
miR-221/222	Thyroid papillary carcinoma, glioblastoma	p27	Increases cell population in the S phase	Cell cycle	Enhances genomic instability	[36,37]
miR-372	Testicular germ cell tumor, cervical cancer	LATS2, CDK2, CCNA1	Compromises p53-mediated CDK inhibition; Delays entrance into G2/M from S phase	Cell cycle	Enhances genomic instability	[38,39]
miR-373	Breast cancer, hepatocellular carcinoma	RAD52 and RAD23B	Impairs HR repair	DNA damage response	Enhances genomic instability	[35]
miR-421	Neuroblastoma	ATM	Impairs response to DNA damage	DNA damage response	Enhances genomic instability	[40]
miR-504	Colon cancer	P53	Reduces p53 expression	DNA damage response	Enhances genomic instability	[41]
miR-1255b	Breast cancer	BRCA1 and BRCA2	Impairs HR repair	DNA damage response	Enhances genomic instability	[31]

Although the roles of miRNAs in other cancer characteristics have been intensively reviewed, miRNA function in genomic instability is relatively less covered. There are several excellent reviews focusing on miRNA function in DNA damage repair [42,43,44,45,46]. In this review, we aim to include also findings on miRNAs affecting cell cycle checkpoint and mitotic separation, and discuss the implications of such findings in the context of cancer (Table 1).

## 2. MiRNAs Are Frequently Located in the Fragile Sites of the Genome

Fragile sites are nonrandomly distributed chromosomal loci that have the tendency to form gaps or breaks. Early in 1984, Yunis and Soreng proposed that changes at constitutive fragile sites might underlie the carcinogenic process of human cancers, based on the positive association between fragile sites and chromosomal rearrangements in cancer [47]. A substantial body of literature has supported this notion by demonstrating that fragile chromosomal loci predispose cancer cells to genomic instability, and that alterations at these sites play a causative role in human cancer [48].

Altered miRNA expression profiles have been observed in many types of human cancers. In 2004, Calin *et al.* revealed that miRNA genes are located within fragile sites that are prone to alteration at a rate that was nine times higher than in non-fragile sites [49]. More than half of the 186 miRNAs surveyed in this study were located in fragile sites, indicating a causative role of miRNAs in cancer initiation and progression [49]. Additionally, miRNA gene loci and insertion sites of human papilloma virus strains causing endometrial cancer were significantly associated [49]. This indicates a possibility that viral insertions in the human genome might disturb miRNA expression [50]. Using mouse models of murine leukemia virus infection, Wang *et al.* demonstrated that retrovirus infection induces the expression of the oncogenic miR-17-92 miRNA cluster [51]. Previous studies have revealed that many minimal regions of deletions harbour tumor suppressor miRNAs, and those of amplifications contain oncogenic miRNAs. For instance, the frequently deleted 0.54 mb region at 13q14.3 in CLL patients contains *MIR15A* and *MIR16-1* genes; the chromosomal region 11q23-q24 that is frequently deleted in breast and lung cancers contains genes coding for the tumor suppressor miR-34a; the frequently deleted chromosomal region 9q22.3 in urothelial cancer harbours multiple miRNA genes including miR-24-1/miR-27b/miR-23b and let-7a-1/let-7f-1 [49]. Similarly, the 17q23 genomic region harbouring the miR-21 gene, is frequently amplified in neuroblastoma, and the oncogenic miR-17-92 cluster is amplified in lymphoma [52]. In addition to genomic amplification or deletions, chromosomal translocation or other genomic instability events could also affect miRNA expression. For instance, acute myeloid leukemia with translocation (8;16) (p11;p13) showed a specific miRNA signature containing 94-miRNAs, most of which were epigenetically downregulated [53]. In another report, activation-induced cytidine deaminase (AID) provoked genomic instability in mature B cells, among which are reciprocal translocations causing fusion of miR-142 and MYC, suggesting that AID affects not only immunoglobulin genes, but also non-immunoglobulin genes such as miRNA [54].

The association of miRNA genes with fragile sites of the human genome suggests that miRNA expression could be altered during initial stages of carcinogenesis, and further indicates that miRNAs could be the early events causing genomic instability, and initiating the carcinogenic events.

## 3. MiRNAs in Genomic Instability and Cancer

Genomic instability drives tumorigenesis by enabling the genomes of cancer cells to acquire the changes necessary to escape their normal restraints. The factors causing genomic instability can be categorized into cell cycle defects, mitotic defects, or DNA damage repair regulatory defects. Many miRNAs are altered during carcinogenesis [55,56], and some of these deregulated miRNAs modulate the expression level of proteins in the above biological pathways, and subsequently lead to genomic instability [42].

### 3.1. MiRNAs and Cell Cycle

The cell division cycle contains four phases: in the G1 (G indicating gap) phase, cells increase the number of organelles; during the S (synthesis) phase, DNA replication begins; the G2 phase is characterized by rapid cell growth and protein synthesis; in M (mitosis) phase chromosomes are separated into two identical nuclei, followed by cytokinesis, during which cells are subsequently divided into two daughter cells. Multiple molecular pathways and checkpoints tightly control each cell cycle phase. Cyclin dependent kinases (CDKs), which add phosphate to a protein, along with cyclins act as major control switches regulating the progression through the cell cycle. Cyclin dependent kinase inhibitors, such as p21 and p27, also regulate the cell cycle by affecting CDK functions. Defects in cell cycle control checkpoints could lead to early or delayed entry to the next phase, abnormal genomic contents, and immature chromosome separation, and consequently cause genomic instability and cancer. Many miRNAs have been reported to affect the cell cycle, and here we review several typical examples of such interactions.

#### 3.1.1. MiR-372/LATS2, CDK2, CCNA1

A screening study identified miR-372 as an oncogenic miRNA facilitating cellular transformation in primary cells with oncogenic Kirsten rat sarcoma viral oncogene homolog (KRAS) and wild type p53. Further mechanistic study revealed that miR-372 directly targets LATS2, a protein interacting with mouse double minute 2 homolog (MDM2) and activating p53, and as a result compromises p53-mediated CDK inhibition [38]. LATS2 was shown in another study to form a positive feedback loop with p53 to guard the stability of the chromosome, and prevent the formation of tetraploid cells [57]. Thus, it can be deduced that aberrant miR-372 expression may promote genomic instability, and subsequently lead to oncogenic transformation. Interestingly, in another study on human cervical cancers, miR-372 was shown to exhibit a tumor suppressor role as evidenced by reduced cell proliferation in cells overexpressing this miRNA. This effect was mediated by the effect of miR-372 on CDK2 and CCNA1, and the subsequent paused entrance into G2/M from S phase of the cell cycle [39]. Despite the functional discrepancies, targeting cell cycle checkpoint proteins in both studies suggests a role of miR-372 in cell cycle regulation and genomic instability.

#### 3.1.2. MiR-106b-25 Cluster/p21

Based on the reasoning that expression association of individual miRNAs and mRNA genes enriched in cellular signaling pathways may indicate functional involvement in such pathways, Ivanovska *et al.* classified miRNAs by correlating their expression levels with those of nearly 40,000 mRNA transcripts [26]. Several members of the miR-106b-25 cluster and its paralog miR-17-92 cluster, including miR-106b, miR-106a, miR-20, and miR-17-5p, were associated with DNA replication/mitosis, suggesting a role of these miRNAs in cell cycle regulation. The authors further concluded that overexpression of the miR-106b cluster is a potential contributor to the highly proliferative nature of tumor cells via deregulation of the cell cycle. Additionally, cells transfected with locked nucleic acid (LNA) based anti-miRs designed to suppress endogenous expression of miR-106a, miR-106b, miR-20, and miR-17-5p showed a greater percentage of G1 cells compared to the control cells, suggesting that these miRNAs are required for cell entry from G1 to S phase. After identifying the CDK inhibitor p21 as a target of miR-106b via microarray, the authors established a functional connection by silencing p21 with an siRNA in cells transfected with anti-miR-106b. The previously observed accumulation of cells in G1 was abrogated after p21 silencing, suggesting p21 as an indispensible mediator of the anti-miR phenotype. The regulation of cell cycle checkpoint proteins by the miR-106b-25 cluster indicates an involvement of such miRNAs in genomic instability [26].

#### 3.1.3. MiR-221/222/p27

P27, a cyclin-dependent kinase inhibitor, blocks the activation of cyclin E-CDK2 or cyclin D-CDK4 complexes, and controls cell progression at G1 phase [58]. The modulation of p27 expression by miRNAs could thus affect genomic stability by causing cell cycle defects. The miR-221/miR-222 cluster miRNAs are upregulated in human thyroid papillary carcinoma. Bioinformatics tools predict p27 as one of direct targets for these miRNAs, and luciferase assay validated this interaction. Ectopic expression of miR-221 caused an in increase in the cell population in S phase, following reduced p27 protein expression in these transfected cells [36]. The regulatory effect of miR-221/222 on p27, and subsequent effect on cell proliferation was also demonstrated in another study using glioblastoma cells. In this study, the authors further proved the indispensible role of p27 by showing that p27 inhibition abrogated the growth advantage of cells with miR221/miR-222 downregulation [37].

#### 3.1.4. MiR-24/E2F2, CDK1, CDK4

During cell terminal differentiation such as muscle and neuronal cell differentiation, miR-24 expression was found to be upregulated. Lal *et al.* demonstrated with gene ontology analysis that in miR-24 overexpressing cells, the 248 mRNAs shown to be downregulated were highly enriched in pathways of DNA repair and cell cycle regulation. MYC and E2F2, which play central roles in regulating G1/S transition as well as progression through S phase of the cell cycle, are the major nodes connecting the above-modulated networks [13]. The expression levels of MYC and E2F2, as well as multiple E2F2- and MYC-regulated genes were reduced in miR-24 overexpressing cells. However, knockdown of E2F2 (but not MYC) completely abrogated the proliferative effect of anti-miR against miR-24. Additionally, overexpression of miR-24-insensitive E2F2 restored the proliferative phenotype to miR-24-treated cells, supporting the notion that antiproliferative effect of miR-24 is mediated by its direct regulation of E2F2 [13].

### 3.2. MiRNAs and DNA Damage Response

DNA within the human genome is continuously exposed to various exogenous and endogenous stimuli that have the potential to elicit DNA damage. An evolutionarily conserved DNA damage response detects and repairs DNA damage to ensure the stability of the genome. Defects in the DNA damage response could cause genomic DNA mutations, deletions, insertions, or gross chromosomal gains and losses upon cell division, and subsequently lead to cancer. A large body of literature has reported the involvement of miRNAs in regulating the DNA damage response.

#### 3.2.1. MiR-155/MLH1, MSH2, MSH6, TERF1

DNA mismatch repair (MMR) is a system that recognizes and corrects insertions, deletions, and misincorporated base pairs during DNA replication and recombination, and inactivation of MMR is a cause for a large variety of human cancers. Mutations in the genes coding for MutS homologs and MutL homologs, two essential members of the DNA MMR pathway, often lead to microsatellite instability and cancer. The expression of oncogenic miR-155 is upregulated in many cancer types, and miR-155 alone is sufficient to induce lymphoblastic leukemia [59]. Computational algorithms predicted, and subsequently *in vitro* studies confirmed several essential MMR genes, including *MLH1*, *MSH2*, and *MSH6*, as potential binding sites for miR-155*.* Colon cells overexpressing miR-155 displayed acquired microsatellite instability with downregulated levels of the above MMR core proteins [32]. In a recent paper, Dinami *et al.* showed that miR-155 impairs telomere integrity by downregulating the expression level of telomeric repeat binding factor 1 (TERF1) in human breast cancer [33]. They demonstrated that high miR-155 expression leads to increased genomic instability, and correlates with poor clinical outcome in estrogen receptor-positive breast cancer. Consistently, downregulation of miR-155 expression improved genomic stability by increasing telomere stability. These findings suggest that miR-155 has multiple functions in causing genomic instability [33].

#### 3.2.2. MiR-29/34/122/125b/504/p53 and p53-Regualted Genes

As an important sensor in the DNA damage response, p53 functions to block the cell cycle to repair damaged DNA, or direct cells to apoptosis in cases of severe damage with no potential for repair. In the case of Li Fraumeni syndrome, an inherited *TP53* mutation was found to cause a high risk of cancer in individuals [60]. Several miRNAs affect p53 or p53-regulated genes via different mechanisms. For instance, miR-125b [29] and miR-504 [41] directly target p53 by binding to 3'-UTR of p53, and negatively regulate p53 expression; while miR-29 indirectly upregulates p53 by targeting PIK3R1 and CDC42 [16], and miR-122 increases p53 protein stability via targeting CCND1 [27]. Previous studies revealed that miR-34, a miRNA downregulated in many cancer types, is transcriptionally activated by p53, and elicits various p53 downstream effects by posttranscriptional repression of oncogenes including CCND1, CCNE2, CDK4, MET, MYC, SNAI1, and SIRT1 [17,18,19]. Multiple studies have shown that miR-34 is indispensible for the DNA damage response [20], and pathway analysis showed that miR-34a-regulated genes are highly enriched in signaling of cell-cycle progression, DNA repair, and apoptosis [19]. Because of its consistent tumor suppressor function, miR-34 has been developed into replacement therapy for treatment of advanced hepatocellular carcinoma, and a clinical trial with this agent is ongoing [7].

#### 3.2.3. MiR-182/193b/1255b/BRCA1

The deficiency of BRCA1, an important regulator of DNA damage repair, has been reported to cause abnormalities in the S-phase checkpoint, the G2/M checkpoint, the spindle checkpoint, and centrosome duplication. BRCA1 mutation has been correlated with a higher degree of aneuploidy, such as chromosomal gains and losses, homozygous deletion of tumor suppressors p53 and PTEN, and loss of heterozygosity at multiple loci, when compared to BRCA1 wild type breast cancers [61]. MiR-182 is predicted to target several double stranded break (DSB) repair proteins including BRCA1. In their 2009 study, Moskwa *et al.* determined the interaction of miRNAs and their targets *in vivo*, based on the fact that miRNAs target messenger RNAs with a protein complex including Argonaute proteins AGO1 and AGO2 [34]. They demonstrated that the miR-182/AGO1 complex associates selectively with the BRCA1 RNA transcript by use of immunoprecipitation with HA-tagged AGO1 in miR-182 overexpressing cells. As expected, BRCA1 protein levels were significantly downregulated alongside ectopic overexpression of miR-182, and consequently significantly higher level of residual DNA damage was observed in the overexpressing cells. Finally, the authors proved that the influence of miR-182 on DNA damage response to ionizing radiation is mediated by BRCA1 [34]. Choi *et al.* identified via gain-of-function screen that miR-193b and miR-1255b specifically suppress homologous recombination in the G1 phase by targeting BRCA1 as well as BRCA2, and proposed that these miRNAs maintain genomic stability by preventing homologous recombination in G1 cells, which could induce loss of heterozygosity [31].

#### 3.2.4. MiR-24/138/H2AX

H2AX is a sequence variant of histone H2A that is utilized in DNA repair, and phosphorylation of H2AX is one of the early events in the response to DSB. In their 2009 study, Lal *et al.* showed that miR-24 targets H2AX expression by binding to two sites in its 3'-UTR, and downregulates its expression by promoting mRNA decay and inhibiting translation [14]. Additionally, the authors showed that cells overexpressing this miRNA are hypersensitive to DNA damage by cytotoxic drugs, demonstrating that miR-24 impairs DNA repair by mediating H2AX expression. Irradiated miR-24 overexpression of cells showed two times as many chromosomal breaks and fragments as those transfected with an empty construct, while cells co-transfected with a miR-24 and miR-24-insensitive H2AX plasmid (one lacking the miR-24 binding site) had fully rescued DNA repair, with no significant increase in DNA damage compared to the control group. Conversely, cells with suppressed miR-24 showed significantly enhanced DNA repair, further supporting that H2AX is the key target of miR-24 in cellular DSB response [14]. Similarly, using ionizing radiation-induced gamma-H2AX foci screening, Wang *et al.* identified that miR-138 reduces H2AX expression by targeting its 3'-UTR region [30]. Ectopic expression of miR-138 sensitizes cells to ionizing radiation or DNA-damaging agents such as cisplatin, and camptothecin [30].

#### 3.2.5. miR-16/100/101/421/ATM

Ataxia telangiectasia mutated (ATM) is an important mediator of connecting DNA damage signals to downstream events including damage repair. Several independent reports showed that miR-100 [23], miR-101 [24], and miR-421 [40] could directly target ATM via the canonical action mechanism. In addition, miR-101 also targets PRKDC to regulate non-homologous end joining (NHEJ) of DNA double strand breaks [24]. The early-identified tumor suppressor MiR-16 also regulates ATM signaling indirectly, through targeting the ATM inhibitor WIP1 [12]. Interestingly, in response to DNA damage, more than one-fourth of miRNAs are significant upregulated in ATM-dependent manner [62]. This upregulation was achieved by ATM-induced KSRP phosphorylation, and consequent increased interaction between KSRP and pri-miRNAs, which promotes processing from pri-miRNAs to pre-miRNAs [62]. Although not fully determined, many of these DNA damage-induced miRNAs may play functional roles in DNA damage response.

#### 3.2.6. MiR-96/103/107/148b/193b/RAD51

RAD51 is an essential protein for homologous recombination of DNA during DSB repair. Multiple lines of evidence revealed that miRNAs could target RAD51 to regulate DNA damage response. Neijenhuis *et al.* showed high miR-107 and low RAD51 expression in a subset of ovarian cancer, and demonstrated that miR-107 reduces RAD51 expression [63]; Huang *et al.* identified several miRNAs including miR-103 and miR-107 that are directly involved in regulating RAD51 and RAD51D using miRNA mimics screening [25]; Wang *et al.* showed that miR-96 reduces the levels of RAD51 and REV1 [21]; Choi *et al.* showed that miR-148b and miR-193b reduce RAD51 expression, and suppress the homologous recombination specifically in the G1 phase [31]. In all these studies, reducing RAD51 levels by miRNAs compromises homology-directed repair, and sensitizes cells to DNA damaging therapeutic drugs such as PART inhibitor AZD2281 and cisplatin. The correlation of high miRNA and drug sensitivity also suggests that these miRNAs could be useful biomarkers for distinguishing patients with better drug response. 

#### 3.2.7. MiR-210/373/RAD52

RAD52 is a key factor in homology-dependent repair by stimulating of the RAD51 recombinase. Crosby *et al.* showed that miR-210 and miR-373 expression is induced under hypoxic conditions [35]. Forced expression of either miR-210 or miR-373 reduced the RAD52 expression via direct interaction with 3'-UTR of RAD52. In addition, miR-373 also targets RAD23B, a protein involved in nucleotide excision repair [35]. These findings elegantly demonstrate the importance of miRNAs in connecting hypoxia with DNA damage repair pathways.

### 3.3. MiRNAs and Mitotic Events

Erroneous distribution of chromosomes during mitosis jeopardizes cellular function and often results in chromosomal instability. If the mitotic machinery meant to ensure accurate segregation of genetic material between daughter cells is disturbed or compromised, the potential of somatic cells reaching an aneuploidy status, and consequently initiating carcinogenesis, increases [64]. Recent findings have identified the participation of several miRNAs in the regulation of mitotic events.

#### 3.3.1. MiR-125b/MXD1

The spindle assembly checkpoint is a “wait-anaphase” mechanism and its proper functioning is necessary to ensure that the chromosomes segregate accurately during mitosis. A key event in spindle assembly checkpoint activation is the loading of the MXD1 protein to the kinetochore, where it serves as an adaptor for MAD2L2 transportation from the cytosol into the nucleus in addition to its role in kinetochore localization. Bhattacharjya *et al.* determined that miR-125b negatively regulates MXD1 expression by sequence-specific binding to its 3'-UTR, and miR-125b and MXD1 expression levels are inversely correlated in human cancer cell lines as well as primary head and neck cancer tissues [28]. The consequence of miR-125b regulation on MXD1 is delayed metaphase and elevated chromosomal abnormalities [28].

#### 3.3.2. MiR-100/PLK1

Polo-like kinase 1 (PLK1) is a critical regulator of mitosis and possesses functions including, among others, microtubule nucleation and spindle maintenance. Overexpression of this kinase has been observed in many types of human cancers, and high PLK1 expression is associated with poor clinical outcome [65]. Shi *et al.* demonstrated that PLK1 is a miR-100 target, and underexpression of miR-100 leads to high levels of PLK1, which in turn cause mitotic catastrophe and progression of human nasopharyngeal cancer [22]. Owing to the essential role of PLK1 in genomic instability and carcinogenesis, it has been suggested as a potential therapeutic target. The epigenetic regulation of PLK1 by miR-100 suggests that the inhibition of PLK1 could be achieved through miR-100 replacement.

#### 3.3.3. MiR-24/AURKB

Aurora kinase B (AURKB), a key regulator of mitosis, participates in chromosome segregation via association with microtubules forming the mitotic spindle. Aberrant AURKB expression has been shown to promote genomic instability and carcinogenesis [66]. A recent study showed that ectopic miR-24 expression reduced the protein levels of AURKB in a hepatocarcinoma cell line; however, AURKB levels are not altered by miR-24 in MHH-CALL-3 TCF3-rearranged leukemic cells [15]. Nonetheless, this study suggests that miR-24 could potentially alter expression levels of proteins involved in mitotic separation, and thus play significant roles in genomic instability.

## 4. Conclusion Notes

The two perceptions of miRNA and genomic instability each stand strongly on their own in terms of significance in the realm of cancer research. Impressively, miRNAs as a concept have been discovered, explored, and applied to clinical research in just a matter of 20 years and the body of literatures surrounding both of these topics is large and growing very rapidly. It is clear that their presence in the human genome is often intertwined and that the spectrum of their impact on every part of the tumorigenic process is wide and significant.

It should be noted that although in this review we separated the mechanisms of genomic instability into three categories, these molecular mechanisms are deeply connected and linked to each other. For instance, the proteins involved in cell cycle arrest may also have functional effects in DNA damage response, and indeed cell cycle arrest itself is one of the responses to DNA damage. In addition, a single miRNA may have multiple protein targets participating in genomic instability with different routes. As a typical example, miR-24 can regulate proteins involved in cell cycle checkpoint, DNA damage response and mitotic separation. It can thus be hypothesized that a single event of miRNA change due to genomic deletion, epigenetic regulation, or transcriptional regulation, could affect multiple signalling pathways leading to genomic instability.

The link between miRNA and genomic instability could be significant for translational medicine. First, it is possible to target genomic instability as a prevention strategy with miRNA-based agents. Second, the miRNA involvement in sensitization of cancer cells to chemotherapeutic drugs or radiation suggests a possible combination of miRNA mimics or inhibitors with current therapeutic regimen. Last but not least, miRNAs could be useful biomarkers in identifying cancer patients who may benefit from treatment with PARP inhibitors, and other chemotherapeutic DNA-damaging drugs.
